# Supplementary material for: Assessing genotypes of buffel grass (Cenchrus ciliaris) as an alternative to maize silage for sheep nutrition
Source: PLoS One. 2024 May 24;19(5):e0304328. doi: 10.1371/journal.pone.0304328 (PMC11125467; doi:10.1371/journal.pone.0304328)
Supplement: S1 File — (PDF) [file pone.0304328.s001.pdf]

Table S1-S7: Supplementary Data: Replication Dataset for Final Tables

Table S1: Fresh sample used for in vitro silage (mg/g of dry matter)

**Genotype**	**DM**	**OM**	**EE**	**CP**	**NDF**	**ADF**	**Cellulose**	**Hemicell**	**Lignin**	**Sugar**
IG 96-401-R1	399.40	874.70	18.90	61.00	762.30	469.00	372.10	293.30	56.95	65.91
IG 96-401-R2	381.40	872.60	18.46	51.50	758.50	470.30	369.20	298.20	56.95	80.80
IG 96-401-R3	387.10	873.65	18.69	56.30	777.10	463.70	361.20	305.40	59.85	92.54
IG 96-50 -R1	319.90	855.90	18.30	52.30	789.50	502.10	363.70	297.40	77.02	94.40
IG 96-50 -R2	337.40	854.80	18.50	59.30	789.50	495.30	367.70	284.50	73.05	86.08
IG 96-50 -R3	330.80	857.85	18.40	58.70	795.80	462.90	353.40	332.90	62.30	72.09
IG 96-96-R1	348.60	849.50	15.10	71.10	733.40	519.30	387.30	214.10	82.18	93.20
IG 96-96-R2	351.90	850.72	14.70	65.70	731.70	517.20	384.40	215.20	82.08	81.12
IG 96-96-R3	349.80	848.11	14.90	68.90	735.10	510.70	383.80	218.10	85.01	67.08
IG 96-403-R1	338.00	882.50	16.70	55.10	764.80	458.80	346.80	309.90	64.59	89.44
IG 96-403-R2	328.10	875.20	16.40	59.30	765.80	456.90	347.70	308.80	63.90	58.84
IG 96-403-R3	320.80	888.85	16.55	60.30	768.90	451.10	348.50	316.60	60.98	72.98
IG 97-358-R1	344.10	904.10	15.40	95.10	779.80	444.50	350.50	323.30	70.50	93.33
IG 97-358-R2	355.90	899.80	15.70	91.20	784.10	464.20	364.10	326.90	71.94	79.11
IG 97-358-R3	348.20	899.95	15.55	88.40	752.50	448.10	357.30	305.70	65.20	62.09

Table S2. Carbohydrates, protein fractions and energy value of fresh five Cenchrus ciliaris genotypes

Genotype	TCHO	NSC	Sc	pA	PB1	PB2	PB3	PC	DE	ME	NeL	NEG	NEM	TDN
IG 96-401-R1	800.05	56.15446	743.8955	432.8	114.7	174.1786	59.09359	219.2278	8.076149	6.630518	3.986167	1.090251	4.099851	438.2157
IG 96-401-R2	797.5969	43.81532	753.7816	300.1	229.4	170.8645	115.7581	183.8774	8.206924	6.737884	4.058836	1.176267	4.185867	445.3116
IG 96-401-R3	798.8235	49.98489	748.8386	366.45	172.05	172.5216	87.42585	201.5526	8.141536	6.684201	4.022502	1.133259	4.142859	441.7637
IG 96-50 -R1	801.8	32.86518	768.9348	387.7	122.9	169.1842	84.33601	235.8798	7.046748	5.78538	3.414148	0.413167	3.422767	382.3599
IG 96-50 -R2	813.5	37.16999	776.33	373.1	139.1	166.6873	111.4226	209.6901	8.253715	6.7763	4.084837	1.207044	4.216644	447.8505
IG 96-50 -R3	807.65	35.01758	772.6324	380.4	131	167.9358	97.87931	222.7849	7.650231	6.28084	3.749492	0.810105	3.819705	415.1052

IG 96-96-R1	765.9	56.42071	709.4793	216	311.1	116.847	101.7734	254.2797	6.909974	5.673089	3.338145	0.323204	3.332804	374.9385
IG 96-96-R2	777.524	66.58276	710.9413	373.1	205.4	65.62791	158.0883	197.7838	6.995158	5.743024	3.38548	0.379234	3.388834	379.5606
IG 96-96-R3	771.712	61.50174	710.2103	294.55	258.25	91.23743	129.9308	226.0317	6.952566	5.708057	3.361813	0.351219	3.360819	377.2496
IG 96-403-R1	809.1	63.88649	745.2135	446.4	168	73.81612	135.0354	176.7485	8.323301	6.83343	4.123505	1.252814	4.262414	451.6263
IG 96-403-R2	795.3	45.04814	750.2519	400.8	154.4	132.8298	138.3213	173.6489	8.53446	7.006792	4.240842	1.391703	4.401303	463.0839
IG 96-403-R3	802.2	54.46732	747.7327	423.6	161.2	103.3229	136.6784	175.1987	8.428881	6.920111	4.182174	1.322259	4.331859	457.3551
IG 97-358-R1	790.65	29.95912	760.6909	423.9	76.6	163.6607	132.9151	202.9242	8.407285	6.902381	4.170173	1.308054	4.317654	456.1833
IG 97-358-R2	783.05	60.05461	722.9954	382.9	196.4	79.3614	179.2452	162.0934	8.558456	7.026492	4.254176	1.407486	4.417086	464.3859
IG 97-358-R3	786.85	45.00686	741.8431	403.4	136.5	121.5111	156.0802	182.5088	8.48287	6.964436	4.212175	1.35777	4.36737	460.2846

Table S3. Fermentation quality of silages made using five Cenchrus ciliaris genotype

			(g/kg of dry matter)	
Genotype	Dry matter	pH	Lactic acid	NH3-N
IG 96-401-R1	333.9	4.1	26.93	0.55
IG 96-401-R2	333.9	4.1	27.82	0.41
IG 96-401-R3	343.7	4.0	29.54	0.21
IG 96-401-R4	343.7	4.0	28.10	0.41
IG 96-50 -R1	274.3	4.7	17.28	0.35
IG 96-50 -R2	274.3	4.65	17.15	0.40
IG 96-50 -R3	273.1	4.57	16.82	0.49
IG 96-50 -R4	273.1	4.59	16.98	0.76
IG 96-96-R1	278.5	4.99	14.90	0.70
IG 96-96-R2	258.5	5	13.59	0.74
IG 96-96-R3	247.5	4.97	14.51	0.69
IG 96-96-R4	247.5	4.93	11.83	0.69
IG 96-403-R1	253.1	4.88	12.65	0.56
IG 96-403-R2	253.1	4.86	12.90	0.63
IG 96-403-R3	256.1	4.97	10.62	0.77
IG 96-403-R4	256.1	4.93	9.11	0.98
IG 97-358-R1	263	5.21	10.29	0.96

IG 97-358-R2	261.5	5.12	11.03	0.82
IG 97-358-R3	277.6	5.16	10.72	0.90
IG 97-358-R4	277.6	5.21	11.06	0.96

Table S4. Chemical composition, pH and lactic acid of silages fed to sheep

	DM	OM	CP	EE	NDF	ADF	Cellulose	Lignin	Hemicellulose	pH	LA
IG 96-401 Silage_R1	399.56	855	52.5	17.3	760.2	496.2	364.4	69.5	264	4.49	34.8
IG 96-401 Silage_R2	354.93	874.9	64.7	17.8	769.4	517.7	369	83.4	251.7	4.35	25.15
IG 96-401 Silage_R3	353.3	864.95	58.6	17.55	764.8	506.95	366.7	76.45	257.85	4.37	33.5
IG 96-96 Silage_R1	255.19	879.8	71.7	14.7	783.3	544.9	406.3	83.8	238.4	4.96	25.34
IG 96-96 Silage_R2	315.5	881.6	69.7	15.4	781.8	542.2	389.4	85.5	239.6	4.65	19.62
IG 96-96 Silage_R3	315.5	880.7	70.7	15.05	782.55	543.55	397.85	84.65	239	4.98	17.89
Maize Silage_R1	407.9	920.2	51	21	802.7	534.8	430.4	77.5	267.9	4.79	26.14
Maize Silage_R2	328.9	918.7	67.7	19.7	757.5	480.8	373.6	75.8	276.7	4.59	20.17
Maize Silage_R3	336.9	919.45	59.35	20.35	780.1	507.8	402	76.65	272.3	4.98	24.87

Table S5. Carbohydrates, protein fractions and energy value of silages fed to sheep

	TCHO	NSC	Sc	pA	PB1	PB2	PB3	PC	DE	ME	Nel	NEG	NEM	TDN
IG 96-401 Silage_R1	785.2	45.1451	740.0549	243.7	400.8	44.13827	57.22531	254.1364	7.439072	6.107478	3.632155	0.671216	3.680816	403.6476
IG 96-401 Silage_R2	792.4	46.52727	745.8727	250.3	341.6	44.46367	103.8379	259.7985	6.923172	5.683924	3.345479	0.331885	3.341485	375.6546
IG 96-401 Silage_R3	788.8	45.83619	742.9638	247	371.2	44.30097	80.53159	256.9674	7.181122	5.895701	3.488817	0.501551	3.511151	389.6511
IG 96-96 Silage_R1	793	37.00696	756.393	302.7	213.1	98.1604	78.0396	308	6.270498	5.148079	2.9828	-0.09741	2.912192	340.2402
IG 96-96 Silage_R2	796.5	40.12694	756.3731	354.3	239.1	41.7946	49.77205	315.0333	6.335285	5.201269	3.018801	-0.05479	2.954805	343.7556
IG 96-96 Silage_R3	794.75	38.56695	756.3831	328.5	226.1	69.9775	63.90583	311.5167	6.302891	5.174674	3.0008	-0.0761	2.933499	341.9979
Maize Silage_R1	848.2	69.37696	778.823	387	232.7	27.61233	75.79105	276.8966	6.512851	5.34705	3.117471	0.061998	3.071598	353.3904
Maize Silage_R2	831.3	99.23082	732.0692	381	188.5	54.86005	107.562	268.0779	7.808601	6.410861	3.837495	0.914272	3.923872	423.6984
Maize Silage_R3	839.75	84.30389	755.4461	384	210.6	41.23619	91.67653	272.4873	7.160726	5.878956	3.477483	0.488135	3.497735	388.5444

Table S6. Nutrient intake, digestibility, nitrogen balance and nutritive value of the silage diets in sheep

	**Nutrients intakes**									
Silage	Animal weight (kg)	DM (g/day)	DM (% body weight)	Dry matter (g/kgw0.75)	Crude protein (g/day)	Crude protein (g/kgw0.75)	Digestible CP (g/day)	Digestible CP (g/kgw0.75)	ME (MJ/day)	ME (MJ/kgw0.75)
IG 96-401 _R1	17.30	505.07	2.92	59.54	57.93	6.83	31.57	2.45	6.21	0.43
IG 96-401 _R2	24.70	682.12	2.76	61.57	69.99	6.32	29.65	1.61	4.48	0.48
IG 96-401 _R3	22.90	648.21	2.83	61.92	67.68	6.47	37.48	2.18	5.57	0.57
IG 96-401 _R4	25.20	670.82	2.66	59.64	69.22	6.15	35.03	1.72	5.16	0.61
IG 96-96_R1	22.50	696.91	3.10	67.46	72.73	7.04	35.79	2.34	5.88	0.53
IG 96-96_R2	22.40	717.14	3.20	69.65	74.14	7.20	31.39	1.75	5.92	0.67
IG 96-96_R3	21.50	719.50	3.35	72.06	74.31	7.44	42.41	3.21	6.49	0.57
IG 96-96_R4	23.50	762.33	3.24	71.42	77.30	7.24	41.80	2.87	5.22	0.52
Maize_R1	18.50	651.15	3.52	73.00	62.10	6.96	27.34	1.83	6.86	0.65
Maize_R2	24.70	772.91	3.13	69.76	68.07	6.14	33.10	2.00	5.98	0.70
Maize_R3	23.60	803.35	3.40	75.03	69.56	6.50	29.22	1.57	6.81	0.61
Maize_R4	24.10	829.98	3.44	76.31	70.86	6.51	31.44	1.76	6.85	0.60

	**Nutrient digestibility (g/kg of dry matter)**						**Nitrogen Balance (g/day)**					**Nutritive value**		
Silage	DM	OM	CP	NDF	ADF	Cellulose	N intake	Faecal N	Urinary N	N absorbed	N retention	DE (MJ/kg of DM)	ME (MJ/kg of DM)	Digestible CP (%)
IG 96-401 _R1	567.07	602.88	545.01	545.31	487.82	610.88	9.27	4.22	0.87	5.05	4.18	11.17	9.17	6.25
IG 96-401 _R2	452.71	490.47	423.60	415.91	365.21	492.31	11.20	6.45	1.61	4.74	3.14	9.14	7.50	4.35

IG 96-401 _R3	526.12	562.50	553.84	492.65	438.83	375.75	10.83	4.22	1.35	6.61	5.27	10.44	8.57	5.78
IG 96-401 _R4	552.04	551.43	506.13	509.61	420.22	525.37	11.07	5.47	1.92	5.61	3.69	10.90	8.95	5.22
IG 96-96_R1	459.28	500.02	492.18	422.71	374.37	545.40	11.64	5.91	2.48	5.73	3.24	9.26	7.60	5.14
IG 96-96_R2	482.66	529.76	423.35	482.95	387.44	593.32	11.86	6.84	1.72	5.02	3.31	9.67	7.94	4.38
IG 96-96_R3	563.54	602.45	570.77	553.48	511.05	440.24	11.89	5.10	1.96	6.79	4.82	11.10	9.12	5.89
IG 96-96_R4	463.22	494.41	540.68	438.15	351.37	552.66	12.37	5.68	2.58	6.69	4.10	9.33	7.66	5.48
Maize_R1	523.59	549.53	440.18	496.37	444.37	605.40	9.94	5.56	1.36	4.37	3.01	10.40	8.53	4.20
Maize_R2	556.47	579.23	486.23	544.81	568.44	516.32	10.89	5.60	1.16	5.30	4.14	10.98	9.01	4.28
Maize_R3	537.57	560.31	420.03	537.26	467.05	395.24	11.13	6.45	1.42	4.67	3.25	10.64	8.74	3.64
Maize_R4	513.83	538.42	443.61	515.33	368.37	532.66	11.34	6.31	1.01	5.03	4.02	10.22	8.39	3.79

Table S7. Rumen metabolites and microbial counts of rumen liquor of sheep fed different silages

	pH	Total volatile fatty acids	NH3-N (mg/100 ml)	Total-N (mg/100 ml)	Total Protozoa	Holotrichs	Entodiniomorphs	Fungal sporangia
IG 96-401 Silage_R1	6.89	53.5	20.5	65.01	2.75	0.29	1.90	0.28
IG 96-401 Silage_R2	6.58	105.5	28	41.14	1.94	0.35	2.01	0.23
IG 96-401 Silage_R3	6.64	113.5	23.8	65.06	2.44	0.28	2.05	0.25
IG 96-401 Silage_R4	6.53	70	18.2	53.13	3.12	0.37	3.01	0.26
IG 96-96 Silage_R1	6.84	74	26.6	69.22	3.5	0.39	3.63	0.25
IG 96-96 Silage_R2	6.84	107.5	32.2	72.33	2.75	0.47	3.63	0.19
IG 96-96 Silage_R3	6.9	68	16.8	62.08	3.87	0.36	1.33	0.22
IG 96-96 Silage_R4	6.87	72.5	25.34	51.07	2.37	0.35	2.35	0.20
Maize Silage_R1	6.99	56	21	64.82	3.12	0.45	3.23	0.26
Maize Silage_R2	6.64	78.5	21.4	58.82	4.18	0.35	3.42	0.41
Maize Silage_R3	6.66	117.5	30.8	52.91	3.69	0.41	3.19	0.21
Maize Silage_R4	6.46	97	15.4	54.61	2.25	0.46	1.90	0.29
