# Supplementary material for: Assessing genotypes of buffel grass (Cenchrus ciliaris) as an alternative to maize silage for sheep nutrition
Source: PLoS One. 2024 May 24;19(5):e0304328. doi: 10.1371/journal.pone.0304328 (PMC11125467; doi:10.1371/journal.pone.0304328)
Supplement: Attachment — Submitted filename: cooments PON journal.docx [file pone.0304328.s002.docx]

After reviewing the paper “Assessing genotypes of buffel grass (Cenchrus ciliaris) as an alternative to maize silage for sheep nutrition," The research idea is excellent, particularly because it addresses the issue of feed shortages in animal production. Furthermore, it discusses an important point: which breeding varieties can be ensiled without the use of silage additives. The authors make several interesting points, making it difficult for the reader to follow their main argument. I recommend that the authors identify their main argument and structure their manuscript accordingly.

**Materials & Methods**

1. Material and method are unorganized, author should be organized as follows:

**2.1. Experiment 1: Screening assays for different genotypes** Cenchrus ciliaris

2.1.1. Fresh raw materials

The Experimental location and establishment section is listed

2.1.2. Silage perpration

The author should list the method of preparing silage in the laboratory for five types of feed Following that, it should mention how silage is prepared for sheep in vivo digestibility.

**2.1.3. Fermentation profiles and chemical analysis**

- The author should describe the steps taken to prepare the silage extract, which is estimated in terms of silage pH and lactic acid levels. In terms of lactic acid estimation, we must shorten this wholesale preparation method with lactic acid, which was determined using a spectrophotometric method as described in (reference).
- The chemical composition of fresh and silage, with a brief mention of sugar contents, should be written as follows: Sugar content was determined using... according to (reference). Additionally, include carbohydrate and protein fractions in this section.
  2.2. **Experiment 2: Invivo digestibility by sheep**
- The corn silage used has no memory of its origins or whether it worked with the experiment treatment.
- Rumen metabolites should be referred to as rumen fermentations, and line 224 should be moved to line 227, following line 145.
- The method for estimating volatile fatty acids, ammonia, and nitrogen is not mentioned or referenced. For example, when estimating VFA concentration, did you evulate GC?

2. Some errors must be reconsidered; for example, the author estimates dry matter at 100 °C for 72 hours. It is well known that the relative humidity of air-dried materials is estimated at temperatures of 105°C for six hours, 125°C for four hours, or 135°C for three hours, whereas the total humidity of juicy and green materials is estimated at 40-60°C for 24-72 hours. Furthermore, the estimate of total carbohydrates is supposed to be called nitrogen-free extract; it is known that it is estimated at 100- (moisture + protein + fat + fiber + ash), and non-carbohydrate fiber at 100- (moisture + protein + fat + NDF + ash). This is something that the author must correct.
3. Calculations for energy and digestible crude protein values must be combined with statistical analysis.

**Results**

1. Does Table 1 show the chemical composition of a fresh or silage sample?

It should be clarified in the title of the table and the text, even if it is a fresh sample, where the chemical analysis of the silage sample is, and vice versa?

The same trend with carbohydrates, protein fractions, and energy values in Table 2. Chemical analysis is required for five types of samples, whether silage or fresh. Why does the author review the silage analysis of selected genotype results again in the in vivo assessment? It is a salvation that was discussed during the initial evaluation. I recommend removing it from the in vivo evaluation. Thus, I recommend that the author organize the results as follows:

- Chemical composition of fresh material
- Fermentation quality and chemical composition of silage
- In vivo digestibility by sheep

2. To combine silage fermentation characteristics and chemical analysis, it is advisable to present them in a single table. When writing about materials and methods, arrange them in the order of the table. It is preferable to start with the fermentation characteristics of silage and then include the chemical analysis in a table and write them in a manuscript. This approach will facilitate the interpretation of the results.
3. The results of the evaluation silage of five genotypes show that silage with IG 96-401 had the highest lactic acid, lowest ammonia concentration, and lowest pH. It was preferable to test it in vivo exclusively with maize silage. On what basis did the author select 96-96? Although the silage evaluation indicates that it is the best IG 96-50 after IG 96-401.
4. Why does the author include corn silage in the initial assessment of five types, especially since his main goal is to use it as an alternative feed to corn silage? His genotype selection was supposed to be based on the closest assessment of corn silage as the standard.

**Discussion**

1. In the section of the discussion, the results must be discussed as follows:

- Chemical composition of fresh material

The author should review studies that agree or disagree with the study of chemical composition of fresh sample.

- Fermentation quality and chemical composition of silage
- As you discuss the fermentation parameters for silage, you must clarify whether each parameter in the study conforms to the specifications of good silage or not. For example, if the values of the PH of silage, lactic acid, and ammonia for the standared (Maize silage) and genotypes are recommended for the good silage and indicative of its increase or decrease than the values recommended, you must discuss, with references. Furter, auther sould discuss change chemical composition of silage (labotary ensiling).
- The author should discuss the reasons for his choice of the two types of genotypes for animal evaluation.
- In vivo digestibility by sheep
- It should be noted that if there are results that do not have an impact, such as in nutrient digestion, for example, it is essential to refer to references that either agree or disagree with the study and explain the reasons behind this discrepancy.
- The author cites plants in the discussion that have nothing to do with the plant under study (underlines 459, 460, 464, and 465); however, he must also cite references to the plant under study.
