# Supplementary material for: Assessing genotypes of buffel grass (Cenchrus ciliaris) as an alternative to maize silage for sheep nutrition
Source: PLoS One. 2024 May 24;19(5):e0304328. doi: 10.1371/journal.pone.0304328 (PMC11125467; doi:10.1371/journal.pone.0304328)
Supplement: Attachment — Submitted filename: Response to Reviewers-Final.docx [file pone.0304328.s004.docx]

Dear Editor,

We would like to thank you again for all reviewers and academic editors for their highly insightful comments, which significantly helped me in the improvisation of the quality of our article. I consider the point raised by you for thoroughly reviewing for English language.

In the following pages, I have written the responses point by point for each comment of reviewers. Hopefully, I have incorporated reviewers all comments and advice.

Thanks, and kind regards,

Pushpendra

Response to Reviewers

**Reviewer# 1**

After reviewing the paper "Assessing genotypes of buffel grass (Cenchrus ciliaris) as an alternative to maize silage for sheep nutrition," the author followed all existing comments with one minor change: the paragraph for Rumen fermentations and microbial counts must be moved from line 165 to 170, after line 210.

*Response: Changes are accepted and modified into the manuscript.*

**Reviewer #2:**

Line 114 rephrase the sentence… mixture samples were analyzed as described (14)

*Response: Sentence is rephrased as per the suggestion.*

Line 129 correct the sentence…Total carbohydrate (g/kg of dry matter) of green (fresh) and silage samples were determined

*Response: Sentence is corrected and rephrased.*

Lines 167-168 correct the sentence… entodiniomorphs were identified by the method of Ogimoto and Imai (25)

*Response: Sentence is corrected.*

Line 222- 224: correct the reference style…. Fonnesbeck et al. (1984); Khalil et al. (1986).

*Response: the reference style corrected for both.*

Table 1. revise the hemicellulose value… 2158

*Response: Oh! thanks for notifying. that was typo error, now rectified.*

Line 360. Correct English…An another

*Response: Checked for English language.*
